# *Porphyromonas* *gingivalis* Virulence Factors and Clinical Significance in Periodontal Disease and Coronary Artery Diseases

**DOI:** 10.3390/pathogens11101173

**Published:** 2022-10-11

**Authors:** Lorena Horvat Aleksijević, Marko Aleksijević, Ivana Škrlec, Marko Šram, Miroslav Šram, Jasminka Talapko

**Affiliations:** 1 Faculty of Dental Medicine and Health, Josip Juraj Strossmayer University of Osijek, 31000 Osijek, Croatia; 2 Faculty of Medicine, Josip Juraj Strossmayer University of Osijek, 31000 Osijek, Croatia; 3 Department of Cardiology, Clinical Hospital Center Osijek, 31000 Osijek, Croatia

**Keywords:** atherosclerosis, cardiology, periodontitis, *Porphyromonas gingivalis*, virulence factors

## Abstract

*Porphyromonas gingivalis* is a gram-negative, anaerobic bacterium that lives in the oral cavity. It is an integral part of the oral microbiome, which includes more than 500 types of bacteria. Under certain circumstances, as a consequence of virulence factors, it can become very destructive and proliferate to many cells in periodontal lesions. It is one of the causative agents present extremely often in dental plaque and is the main etiological factor in the development of periodontal disease. During various therapeutic procedures, *P. gingivalis* can enter the blood and disseminate through it to distant organs. This primarily refers to the influence of periodontal agents on the development of subacute endocarditis and can facilitate the development of coronary heart disease, atherosclerosis, and ischemic infarction. The action of *P. gingivalis* is facilitated by numerous factors of virulence and pathogenicity such as fimbriae, hemolysin, hemagglutinin, capsules, outer membrane vesicles, lipopolysaccharides, and gingipains. A special problem is the possibility of biofilm formation. *P. gingivalis* in a biofilm is 500 to 1000 times less sensitive to antimicrobial drugs than planktonic cells, which represents a significant problem in the treatment of infections caused by this pathogen.

## 1. Introduction

Periodontitis is one of the most common inflammatory diseases affecting all age groups, but it has a higher prevalence in the elderly population [1,2]. Periodontitis has a prevalence rate of about 50% worldwide and most commonly occurs in adulthood. The prevalence of severe forms is about 10% which increases significantly between the third and fourth decades [3]. It is defined as a chronic inflammatory disease of the supporting tissues of the teeth of infectious origin [4,5,6]. Supportive tissues of teeth affected by inflammation are the gingiva, periodontal ligament, and the alveolar bone surrounding the tooth [5]. In addition to leading to tooth loss, it has been linked to numerous systemic diseases such as diabetes, atherosclerosis, rheumatoid arthritis, Alzheimer’s disease, gastrointestinal diseases, and even low birth weight in newborns [2,4,7].

Periodontitis occurs as an inflammation of the gingiva associated with the accumulation of dental plaque, which progresses to inflammation of the bone and periodontal ligament, creating so-called periodontal pockets, which are the main feature of this disease [5,6,8].

The polymicrobial synergy and dysbiosis model explains the etiology and pathogenesis of periodontal disease. Bacterial communities are under the control of the host’s inflammatory response in a healthy periodontium. However, after the colonization of subversive bacteria, such as *P. gingivalis*, there is a synergistic increase in the pathogenic potential of the bacterial community, i.e., dysbiosis, due to which the host’s immune response becomes ineffective and destructive to host tissue [9]. Based on their properties and pathogenicity, the main causative agents involved in the development of periodontitis are organized into interconnected complexes [3]. Early colonizers can adhere to the envelope and include bacteria from the yellow (members of the genus *Streptococcus*), green (includes *Aggregatibacter actinomycetemcomitans* serotype a, *Capnocytophaga*, *Campylobacter*, and *Eikenella corrodens*), and purple complexes (*Actinomyces odontolyticus* and *Veillonella parvula*). After determining the early colonizers, the bacteria that make up the orange complex appear, and they are classified as moderate pathogens such as *Fusobacterium nucleatum* and *Prevotella intermedia* [10].

On the other hand, bacteria of the red complex are highly pathogenic and include *Tannerella forsythia*, *Treponema denticola*, and *P. gingivalis* [11]. The main pathogens involved in the development of periodontitis are *Tannerella forsythia*, *Treponema denticola*, and *Porphyromonas gingivalis* [2,4,7]. In addition, acute periodontitis is associated with increased colonization of *A. actinomycetemcomitans* and *P. gingivalis* [1,5].

Patients with chronic periodontitis who smoke have poorer periodontal status, more significant tooth loss, and poorer response to periodontal therapy than non-smokers [5,12]. Diabetes mellitus is a metabolic disorder characterized by hyperglycemia that is highly associated with periodontitis, so periodontitis is noted as the sixth complication of diabetes [5,6,13]. Periodontitis and more severe forms of periodontitis occur more often in people with diabetes than in healthy individuals [5,6]. Poorly controlled diabetes affects periodontitis’ more severe clinical features and vice versa [5,6,13]. It is assumed that immunosuppressive conditions such as HIV infection favor the development of more severe forms of periodontitis [5]. Finally, genetic predisposition in combination with microorganisms leads to the emergence and progression of periodontitis [5,14].

Several factors are involved in the pathomechanism of periodontitis, such as dental plaque, microbiological biofilm, dental plaque calcification, immune response to biofilm, and genetics [5]. The red bacterial complex is strongly associated with advanced periodontitis [15,16,17].

Cardiovascular diseases are widespread in the general population, and coronary artery disease is the leading cause of death, especially in developed countries [18]. Globally, cardiovascular diseases are responsible for 17.9 million deaths worldwide, and in Europe, for 3.9 million deaths. Periodontitis, as well as cardiovascular diseases, belongs to non-infectious diseases (NCDs). It is the 6th most common human disease and affects 740 million people worldwide. It has a prevalence of 45% to 50%, while the most severe form affects 11.2% of the world’s population [19,20]. An increasingly frequent focus of researchers is the influence of periodontal diseases on developing cardiovascular diseases. This is because periodontal diseases are a potential risk factor that can initiate the development, maturation, and instability of atheroma in the arteries [20]. It is presumed that there is a direct and indirect influence of periodontitis on the development of cardiovascular diseases, so it is considered that periodontal pathogens directly reach the bloodstream. Another assumption is that they act indirectly by increasing the systemic level of inflammatory mediators [20].

The transition from gingivitis to periodontitis begins with the appearance of a specific group of oral pathogens, among which *P. gingivalis* has a significant place [21]. Namely, *P. gingivalis* is present in small numbers in the healthy oral cavity. *P. gingivalis* uses iron from the blood in inflamed gums, which activates it. Once initiated, *P. gingivalis* can actively contribute to worsening periodontal damage [22]. Accordingly, the biofilm’s bacterial composition has qualitative and quantitative changes. As a result, the symbiotic relationship between the host and its resident microbiota is disrupted, which causes changes in the host’s immune response. These changes in the host’s immune response represent a defense mechanism and a response to the degradation of periodontal tissue [23]. A characteristic inflammatory response in periodontal tissue involves the production of enzymes and proinflammatory mediators such as matrix metalloproteinase (MMP), interleukin (IL)-1β, IL-6, tumor necrosis factor (TNF)-α, and C-reactive protein (CRP). Following these mediators’ increase, periodontal tissue destruction rates also increase [24]. Thus, in periodontal pockets, there is an increase in the level of inflammatory cytokines, which is a possible risk factor for the development of atherosclerotic cardiovascular disease [20].

The aim of this article is to present the most important virulence factors of *P*. *gingivalis* that affect periodontal and cardiovascular diseases.

## 2. *Porphyromonas gingivalis* (*P. gingivalis*)

*P. gingivalis* is one of the main causative agents found in subgingival plaque patients with periodontitis. It is thought to play a significant role in the pathogenesis of periodontitis [2,25,26]. The already mentioned red bacterial complex, consisting of *P. gingivalis*, *T. denticola*, and *T. forsythia*, is strongly associated with the progression of periodontitis [15,16,17]. Namely, the bacterial species within the consortium, called the red complex, interact. *P. gingivalis* and *F. nucleatum* are physically associated with biofilm formation. The LrrA protein binds human epithelial cells to *T. forsythia* [10]. *P. gingivalis* fimbriae mediate attachment to host cells and participate in the interaction (coaggregation) between several different plaque-forming bacterial species (*A. naeslundii*, *S. gordonii*, and *S. oralis*) [21]. *P. gingivalis* and *T. denticola* have a symbiotic relationship, due to which they use nutrients and show a strong synergy in growth. Topologically, they are closely connected in the biofilm [27]. Since virulence and biofilm formation occur in parallel with clinical signs of periodontal destruction, they are classified as pathogens involved in the clinical destruction of periodontal tissues [23].

*P. gingivalis* can adhere to the host cell’s surface and be incorporated into phagosomes. After cellular autophagy, replication is enabled, while at the same time, cell apoptosis is disabled. In addition, *P. gingivalis* adapts well to oxidative stress, enabled by its virulence factors [16,28]. It is known that *P. gingivalis* has numerous virulence factors that directly and indirectly destroy periodontal tissue through the mediator of inflammation [2,16,29]. *P. gingivalis* avoids clearance by the immune system, exploits its inflammatory response, invades host cells, and virulence factors allow it to survive longer in adverse conditions, ensuring a long existence within host tissues. These mechanisms of invasion and survival contribute to the resorption of alveolar bone, destruction of other periodontal tissues, and increase the risk of developing systemic diseases associated with periodontitis [25,30,31,32,33,34,35,36,37,38]. In addition, virulence factors affect coaggregation, biofilm formation, and dysbiosis of the oral microbiota [2]. *P. gingivalis* is most associated with chronic periodontitis. Its chronic persistence in the periodontium depends on its ability to evade host immunity without inhibiting the inflammatory response, which also benefits other periodontal bacteria [16].

### 2.1. Virulence Factors

Virulence factors of *P. gingivalis* are fimbriae, hemolysin, hemagglutinins, capsule, outer membrane vesicles (OMVs), lipopolysaccharides (LPS), and gingipains (Table 1). Fimbriae are thin, filamentous structures by most strains of *P. gingivalis*. They extend beyond the outer membrane of *P. gingivalis*, promoting biofilm formation, bacterial adhesion to host cells, and bacterial invasion into cells [2,39,40]. *P. gingivalis* has two types of fimbriae, long and short. Long fimbriae are constructed from FimA protein subunits, while short ones are built from Mfa1 subunits [2,39,41,42]. *P. gingivalis* fimbriae bind to host tissues and cells through various molecules, including proline-rich proteins and glycoproteins, staterin, fibrinogen, fibronectin, and lactoferrin [2,43]. In addition to adhesion to host tissues and cells, fimbriae allow *P. gingivalis* to interact with other oral bacteria and allow biofilm to form [2,44]. Invasion into host cells by *P. gingivalis* is achieved by binding long fimbriae to human GAPDH, triggering the host’s immune response [2,45]. Short fimbriae of *P. gingivalis* bind to SspA and SspB proteins of *S. gordonii* but not to their antigenic complex [2,46]. The long fimbriae of *P. gingivalis* can adhere to hydroxylapatite and oral epithelium [47]. They bind to TLR2 and, thus, trigger the inflammatory response by activating and increasing the expression of proinflammatory cytokines such as interleukins, especially IL-8, TNF-α, and NF-κB, involved in the process of bone resorption [2,40]. Moreover, with the help of long fimbriae, *P. gingivalis* achieves resistance to the host’s defense against gram-negative bacteria through the complement system [2]. Furthermore, short fimbriae help differentiate osteoclast precursor cells into osteoclasts and enhance bone resorption by producing IL-1b, TNF-α, and IL-6 [40].

*P. gingivalis* requires heme as a source of iron and protoporphyrin IX in order to survive within the host cell and establish infection. Heme is located within the pigment on the cell surface of *P. gingivalis* in the form of µ-oxo bisheme. µ-oxo bisheme can be formed from heme in two ways. Heme from hemoglobin could react with molecular oxygen and other oxygen types or be formed from hematin molecules [48,51,52]. Heme sources are hemoproteins in saliva, gingival fluid, and erythrocytes. The main mechanisms by which *P. gingivalis* supplies heme via hemagglutinin, hemolysin, and gingipains can also use heme acquisition systems of other bacteria [48]. The availability of heme greatly affects the growth and morphology of *P. gingivalis* and, thus, its virulence. Cells grown under heme deficiency were in the shape of cocobacilii with few fimbriae. Still, they had many extracellular vesicles, while cells grown with excess heme are cocci with more fimbriae but fewer extracellular vesicles [48,53]. Heme also directly increases virulence through the increased production of cytotoxins, acids, and proteases [48,53,54,55]. Heme also affects the ability to bind further heme and the structure of lipopolysaccharides [48,56,57]. *P. gingivalis* cells grown in the presence of µ-oxo bisheme have a layer of dimeric heme on their surface, which protects them from H_2_O_2_, which helps the cell survive in the presence of neutrophil-releasing H_2_O_2_, extracellularly or intracellularly. Accumulation of heme serves as a barrier for other oxidants. *P. gingivalis* grown under excess heme accumulates more FePPIX [48,57,58,59]. *P. gingivalis* requires heme to stimulate growth and virulence, but also, an excessive amount of heme can adversely affect the cell, especially in the presence of excessive proteolysis. Heme can be released from hemoglobin by the proteolytic action of Kgp [48,60,61,62]. The formation of µ-oxo bisheme and its maintenance in this form is promoted by alkaline conditions, and *P. gingivalis* is known to have a slightly alkaline pH optimum growth in inflamed periodontal pockets, which are also alkaline [48]. The heme-binding domains (HA2) on gingipains, Kgp and HRgpA, and the adhesion domains on the hemagglutinin surface, HagA, can bind both heme and hemoglobin. HA2, Kgp, and HRgpA promote µ-oxo bisheme aggregation. *P. gingivalis* can agglutinate and hemolyze erythrocytes. Its haemagglutinating activity is found in fimbriae, haemagglutinins, and gingipains [48,63]. Iron is used more from hemoglobin than other sources [48]. Free heme may be derived from hemoglobin due to its release from erythrocytes degraded in the periodontal pocket by hemolysins and other proteases during gingival bleeding [48,63]. After hemoglobin, albumin is the most abundant protein that carries heme without bleeding [48].

The bacterial capsule is an outer structure that, like an envelope, encloses a bacterial cell. It is built of polysaccharides and water and serves for bacterial survival in adverse conditions [2]. The *P. gingivalis* capsule is also known as the K-antigen and serves as a bacterial cell defense mechanism against phagocytosis and intracellular death [2,29]. The chemical composition of the capsule varies with different strains of *P. gingivalis*, where they can be divided into different K-serotypes if they have a shell or can be complete without a capsule [2,64]. Encapsulated *P. gingivalis* shows a higher survival rate, due to its resistance to phagocytosis by macrophages and dendritic cells, than those without capsules. However, different serotypes of encapsulated *P. gingivalis* also showed various adhesion abilities. Better adhesion to gingival epithelial cells (GECs) is achieved by non-enveloped serotypes, while enveloped serotypes such as K5 and K6 can bind to periodontopathogens such as *F. nucleaotum* via a capsule. The K1 serotype cannot bind by capsule but is most successful in stimulating cytokine and chemokine production. Encapsulated W50 is the most resistant to the bactericidal activity of antimicrobial peptides compared to encapsulated type [2,29,65,66,67,68,69,70]. The capsule of *P. gingivalis* has a vital role in promoting the bacterium’s survival inside the host cells, helping to avoid the activation of the host’s immune system and increasing virulence. Therefore, that is its main determinant of virulence [70]. Moreover, in experimental infections in a mouse model, encapsulated strains of *P. gingivalis* were significantly more virulent than strains without capsules. Precisely, encapsulated strains cause phlegmonous invasive infections that spread after subcutaneous inoculation in experimental animals, while strains without capsules cause non-invasive, localized abscesses [71].

**Figure 1:**
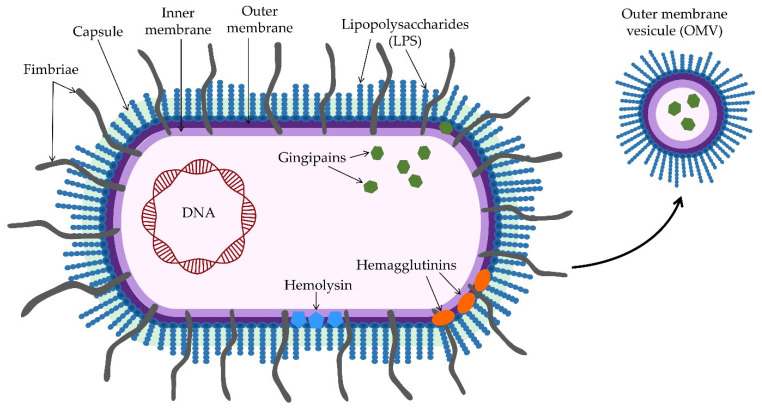
Structure of *Porphyromonas gingivalis* and the most important virulence factors.

Gram-negative bacteria, during their growth, release outer membrane vesicles (Figure 1) while maintaining the integrity of the outer membrane [49]. OMVs consist of outer membrane proteins, phospholipids, lipopolysaccharides, periplasm parts, DNA, and RNA [49,72,73]. Vesicles are involved in bacterial adaptation to stress, nutrient acquisition, and maintaining communication with other bacteria and host cells [49]. They are also responsible for invading host cells and destroying them through pathological mechanisms of avoiding host immune defenses and antibiotic resistance [49,74]. Various mechanisms can form outer membrane vesicles. One of the formation mechanisms is during the growth of the bacterial cell. Due to growth, the bacterial cell wall is stimulated, and muramyl peptides are released, which, if not absorbed, are generated on the outer membrane and later released [49,75]. Vesicles are formed where the bond between the peptidoglycan and the outer membrane is weakened. OMVs are accumulated along the outer membrane, containing virulence factors more strongly than in the parent bacterial cell [49,76]. OMVs can eliminate unwanted substances that accumulate under the influence of stressors and toxins [49], carry antigenic substances for the bacterium’s interaction with host cells and drugs, and thus increase the viability of the bacterium [49,77]. OMVs penetrate better into deep tissues and activate the host’s inflammatory response there, but their virulence depends on the amount of lipids, proteins, and nucleic acids [49,78]. Most *P. gingivalis* OMV proteins are membrane proteins, lipoproteins, and extracellular proteins. OMVs also contain noncoding RNAs and lipoproteins that bind heme. *P. gingivalis* OMVs can return heme-filled OMVs to the biofilm and provide other bacteria in the plaque with nutrients, allowing other species to reproduce. Other bacteria may also use oligosaccharides, monosaccharides, peptides, and amino acids produced by hydrolases in OMVs [49,79,80]. FimA and Mfa1 found in OMVs serve to communicate *P. gingivalis* with other bacteria. They have excellent communication with *S. aureus* and can inhibit other bacteria in the biofilm, such as *S. gordonii*, creating a favorable environment for *P. gingivalis* [49,81]. Small RNAs within OMVs are considered mediators of host–guest communication due to their ability to modulate gene expression in multiple cells and species, for which they use many regulatory mechanisms. Some of them are the capacity to bind protein targets and modify their function and blockage of the ribosome-binding site. Bacterial sRNAs can be released into the environment and transferred to other microbes and host cells [73]. Specific for periodontal pathogens, msRNAs can deactivate anti-inflammatory host response by deactivating the expression of genes that encode cytokines such as IL-5 and IL-13 [82]. OMVs can be embedded in a cell in two different ways. One way is actin-mediated, where OMVs use host cell receptors to initiate F-actin polymerization and, thus, enter the cell. The second way is by fimbriae and the process of endocytosis, which depends on P13K, Rac1, and GTPase [49,78]. Once they enter the host cell, OMVs can cause enormous damage. They inhibit the proliferation of endothelial cells and fibroblasts and angiogenesis, which slows down the healing process [49]. They activate pathogen recognition receptors (PRRs), leading to cytokine secretion and gingival epithelial apoptosis [72]. They stimulate macrophages to produce large amounts of proinflammatory factors such as interleukins IL-6, IL10, and IL-12p70, TNFα, and IFNb and activate caspase-1 and release LDH, ultimately resulting in cell apoptosis [49,72,83].

The outer cell membrane of gram-negative bacteria, including *P. gingivalis*, consists mainly of lipopolysaccharides (LPS); but for virulence of *P. gingivalis*, the most important is LPS-A. Lipopolysaccharide belongs to the group of microbe-associated molecular patterns known by the abbreviation MAMP [2,84]. The term endotoxin is also used in the literature because of its ability to cause an inflammatory reaction in the host organism [2]. It consists of lipid A, the nuclear oligosaccharide that forms the core of its structure, and the O-specific polysaccharide. [2,85]. LPS is built differently in different bacteria. Structural differences mostly depend on the acyl chains of lipid A fatty acids, and their differences can explain the different mechanisms of host recognition of bacterial cells [2]. The basic chemical structure of LPS is similar to that of other bacteria. However, the structure of *P. gingivalis* LPS shows different acylations, tetra-acylations, and penta-acylations, due to which *P. gingivalis* LPS activates specific signaling pathways and specific immune responses [2,86]. *P. gingivalis* LPS plays a significant role in the pathogenesis of periodontal disease. Virulence factors depend on the structure of lipid A. Host cells recognize the lipid component of LPS and immediately trigger an inflammatory response in periodontal tissue, thus creating favorable conditions for maintaining pathogens and progression of inflammation itself and, therefore, periodontal disease itself [2,87]. Activation of TLR by *P. gingivalis* LPS is still in the research focus due to conflicting research results obtained on animal models. One study showed that TLR2 was most important for alveolar bone loss, while others proved the importance of TLR4, indicating that these receptors play a dominant role in the development of periodontitis [2,88,89]. The binding of LPS to individual TLRs and their activation was determined by the structural differences of the lipid A component of LPS. Structural differences cause different immune responses and the production of proinflammatory cytokines. Penta-acylated *P. gingivalis* LPS1690, when bound to TLR4, increases Lipopolysaccharide-binding protein (LBP) expression in oral keratinocytes and activates NF-κB and p38/MAPK pathways [2,90]. By binding to TLR2 receptors, it significantly increases NF-κB transcription. Unlike LPS1690, tetra-acylated LPS1435/1449 binds more to TLR2 than TLR4 receptors. It more significantly increases the expression of TLR2 on the cell surface of hGF than LPS1690, which in turn increases the expression of TLR4 and MD-2 on the cell surface of hGF. LPS1435/1449 can avoid the inflammatory response of the innate immune system, thus allowing *P. gingivalis* to survive longer within macrophages. LPS1690, on the contrary, induces inflammation and production of IL-1 β and reduces the intracellular survival of *P. gingivalis* [2].

Gingipains belong to the group of cysteine proteinases whose role is the cleavage of peptide and polypeptide bonds and which are located on the cell surface of *P. gingivalis* [2,29]. They make up 85% of the total proteolytic activity of *P. gingivalis* [2,29,50,91]. Gingipains can be divided into arginine-specific (Arg-X) and lysine-specific gingipains [2,29]. Arginine-specific gingipains are responsible for the hydrolysis of peptide bonds of carbonyl groups. They can degrade extracellular matrix components such as integrin–fibronectin-binding factors, cytokines, immunoglobulin, and complement components [29]. There are two types of Arg-X gingipains, RgpA, which contains a proteolytic and adhesion domain, and RgpB, which performs hydrolysis exclusively. Kgp and lysine-specific gingipains have catalytic and adhesion domains [2,29]. In order to avoid unwanted proteolytic activity within the cell, gingipains act like inactive zymogens that require further processes to become mature, functional gingipains [2]. Gingipains participate in biofilm formation to promote coaggregation of periodontal pathogens and adhesion to host tissues. RgpA and Kgp are required for coaggregation with other bacteria [2,92]. RgpA also has a dramatic effect on *P. gingivalis’* interaction with host tissue. It is assumed that the presence of RgpA protease may be correlated with the expression of FimA and RgpB, which is necessary for the maturation of FimA, a component of *P. gingivalis* fimbriae that allows *P. gingivalis* to bind to host cells [2,92,93]. RgpA, besides its involvement in coaggregation with other bacteria and interaction with host tissue, is required for the normal growth of *P. gingivalis* and is involved in the collagen degradation process [93]. Gingipains affect capillary permeability, inhibit blood clotting, and increase bleeding of periodontal tissues [2,29]. Gingipains cleave the bonds of adhesion molecules of gingival epithelial cells and, thus, help penetrate LPS and proteoglycans into periodontal tissue [2,29,94]. Gingipains regulate the immune response and affect the production of immune mediators. They degrade α-defensins and β-defensins, which are essential in pathogen elimination. Gingipains also reduce the expression of CD14 receptors on the surface of macrophages, leading to a reduced response to bacterial infection [2,95,96,97]. They break down the C3, C4, and C5 complement components, reducing bacterial elimination and increasing inflammation. Rpg has a more significant role in the breakdown of complement components than Kgp [2,29]. Impaired complement activity and homeostasis of the host–microorganism lead to periodontal tissue destruction [2].

### 2.2. Biofilm

Biofilm formation is a complex and dynamic process of five phases: binding, microcolony formation, matrix formation, maturation, and dispersion [98].

The first or initial phase is the adhesion of bacteria to surfaces in the oral cavity that are numerous. These can be biotic surfaces such as soft tissues of the oral cavity and other bacteria and abiotic surfaces such as dental implants [99]. Surface structures of *P. gingivalis,* such as fimbriae, lipopolysaccharides, and capsule, play an important role in this phase of adhesion to substrates [40]. Flagella and fimbriae are located in the outer membrane, and bacteria use them to allow negatively charged bacteria to attach to environmental surfaces that are also negatively charged [100]. Long fimbriae of *P. gingivalis* are included in autoaggregation, and their subunits of Fim A fimbriae are essential in the initial binding of bacteria to host cells. At the same time, their loss leads to reduced ability to adhere to human epithelial cells and gingival fibroblasts [101]. In addition, lipopolysaccharides (LPS) are also crucial for the initial phase in the formation of *P. gingivalis* biofilm by the lipid group binding LPS to the outer membrane [102].

After the initial phase, i.e., after binding the first layer to the surface, the biofilm matures, i.e., the biofilm grows into a three-dimensional structure forming microcolonies [103]. These structures have the shape of a mushroom or tower consisting of hundreds of layers [104]. As *P. gingivalis* is an anaerobic bacterium, it occupies deeper layers within the biofilm [105]. In a biofilm, bacteria communicate and have specific tasks [106]. There are also persistent cells at rest characterized by extreme antimicrobial tolerance [107]. Based on testing, it has been shown that persistent cells are not mutations but are phenotypic variants [102].

In the next stage, an extracellular matrix consisting of various natural polymers is formed: proteins, glycoproteins, glycolipids, nucleic acids, and extracellular polysaccharides [108]. Additionally, biofilm channels allow cells to be supplied with water, nutrients, and air, giving them new properties [109]. Quorum sensing (QS) mechanisms enable the control of bacterial population density via extracellular molecular signals. The process is mediated by signaling molecules, autoinducers [110]. Interbacterial signaling pathways regulate bacterial growth, metabolism, biofilm production, the occurrence of virulence, and other aggressive properties concerning the host [111]. Al-2 is very important for forming mixed biofilms of *P. gingivalis* with *S. gordonii* [112]. The positive regulator of *P. gingivalis* biofilm production is the hemin-bound transcriptional regulator Har, which controls the expression of hmuY and Mfa1 [113]. During maturation, in the developed three-dimensional structure that arises during the growth of the biofilm, there are microcolonies within which communication between different species takes place [114].

The dispersion phase is the last phase in which the cells diverge, i.e., the transition from the sessile form of the bacterial population to the mobile one. The genes whose products are flagella proteins are reactivated [115]. Finally, bacteria become mobile again because saccharolytic enzymes are formed that destabilize biofilm polysaccharides. Bacteria are released from the biofilm surface, representing the possibility of creating new biofilms [116].

**Figure 2:**
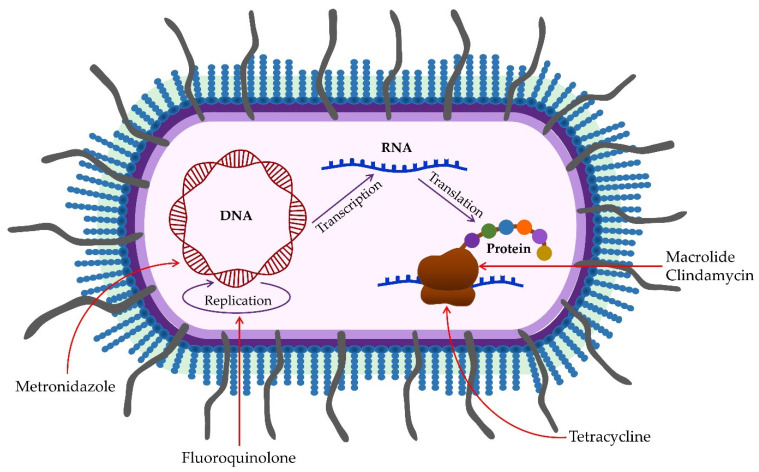
Mechanisms of antibiotic action on *Porphyromonas gingivalis*. Tetracyclines inhibit protein synthesis by binding to the 30S and 50S subunits of the bacterial ribosome. Macrolides and clindamycin inhibit protein synthesis by binding to the 50S subunit of the bacterial ribosome. In contrast, metronidazole and fluoroquinolone inhibit bacterial DNA replication.

Antimicrobial resistance is a growing global problem today [117]. It is also present in treating oral infections caused by *P. gingivalis*. According to available data, it is more pronounced in biofilms [118]. The sensitivity to antimicrobial agents in biofilm is 1000 times less than in planktonic cells [119]. A recent study demonstrated resistance of *P. gingivalis* isolated from patients with peri-implantitis in a percentage greater than 20% to amoxicillin, metronidazole, and clindamycin [120]. These biofilm-induced resistance data suggest the need for new antimicrobial agents [121] (Figure 2).

## 3. Clinical Importance of *Porphyromonas gingivalis* in Periodontal Disease

Periodontal disease represents a group of oral inflammatory infections triggered by oral pathogens found in a biofilm on the surface of the teeth and, under certain circumstances, destroys the tooth’s supporting tissue. The course of the disease ranges from a mild inflammation of the gingiva (gingivitis) to chronic destruction of the connective tissue, followed by the formation of a periodontal pocket and eventual tooth loss [21].

*P. gingivalis* is a significant pathogen of subgingival biofilm [25,26]. In vivo studies in mice have shown that infection with *P. gingivalis* causes bone resorption [122]. *P. gingivalis* adheres to and invades human epithelial cells, where it replicates and causes inflammatory periodontitis [16,123]. Even though the gingival epithelium represents the initial barrier against various pathogens, including periodontal bacteria, *P. gingivalis* invades gingival epithelial cells and underlying connective tissues [25,124,125,126,127]. Moreover, OMV can cause detachment of epithelial cells, which allows breaking down of the initial barrier [25,128]. The mechanisms to decrease E-cadherin expression are *P. gingivalis* LPS, TNF-α, and reactive oxygen species [25,94,129]. Further, both *P. gingivalis* and *P. gingivalis* LPS can cause a loss of transcription factors that regulate junction proteins’ expression in the gingival epithelium [25,130]. *P. gingivalis* fimbriae and the surface of gingival epithelial cells have receptors through which the adhesion of *P. gingivalis* to gingival epithelial cells occurs [25,131,132]. Although gingival epithelial cells are non-phagocytic cells, *P. gingivalis* can induce autophagy in various cells, including gingival epithelial cells (GEC)s. That way, intracellular *P. gingivalis* uses autophagy for its own survival in GECs and replicates in its vacuoles [25,133,134,135,136,137,138]. *P. gingivalis* is most associated with chronic periodontitis [16]. However, it significantly contributes to the pathogenesis of aggressive periodontitis by inducing high levels of proinflammatory cytokines [16,139]. Serotypes K1 and K2 are associated with increased production of RANKL. This factor affects the production and differentiation of osteoclasts and induces a strong Th1–Th17 inflammatory response, resulting in greater resorption of alveolar bone and greater destruction of periodontal tissue [16,140]. Studies in mice infected with *P. gingivalis* showed a significantly higher Treg and Th17 cells level than in the control group [122]. Deep periodontal pockets, severe alveolar bone loss, and bleeding on probing have been associated with increased salivary concentrations of MMP-8, IL-1b, and *P. gingivalis* [16,141]. Higher serum levels of IL-1β, IFNγ, IL-17, RANKL, and MMP-13 were found in mice infected with *P. gingivalis* and *Actinobacillus actinomycetemcomitans* compared to the pristane-induced arthritis group [142].

The complement system protects the host organism from bacterial infection through opsonization, chemotaxis, and lysis of infected cells [25,143]. *P. gingivalis* is resistant to the complement system by its enzymatic activity or through molecules on the cell surface that allow it to adapt to the host’s complement control proteins. This is achieved by binding CD14 to bacterial fimbriae, activating TLR2 receptors and phosphatidylinositol 3-kinase, leading to the conformation of C3 in leukocytes, which induces downregulation of IL-12p70 which is involved in intracellular bacterial clearance [16,144]. *P. gingivalis* prevents the cascade process of C5 convertase formation in the formation of the complement system [25]. Furthermore, C3 in interaction with *P. gingivalis* fimbriae leads to the bacteria’s internalization by macrophages. *P. gingivalis* exploits complement and toll-like receptor synergy and crosstalk to modify host defenses and escape its elimination from the host [16,145]. This is why complement levels in crevicular fluid in patients with periodontitis are low [25,146]. In vivo studies suggested that complement is required in the periodontal disease process. *P. gingivalis* in complement C5a receptor1 deficient mice failed in accomplishing bone loss due to a lack of ability to cause dysbiosis [147,148]. *P. gingivalis* can also avoid killing by neutrophils by decreasing their chemotaxis and reducing their antibacterial capacity [25,149]. Another mechanism for avoiding being killed by neutrophils is via TLR2 and the myeloid differentiation factor 88 (MyD88) pathway in stimulated neutrophils [25,30,38,150], in a way that gingipains remove C5 to produce more C5a to activate C5aR which causes the interaction of TLR2 and C5aR in neutrophils. Their interaction leads to degradation of Myd88, thus interrupting IL-12 release and antimicrobial response [25,38,150]. *P. gingivalis* enhances PI3K signaling, which inhibits G protein RhoA activation and actin polymerization, resulting in decreased phagocytosis of bacteria by neutrophils [25,150]. *P. gingivalis* can also escape NETs killing, structures released by neutrophils during infections, preventing pathogens from disseminating by killing them [25,151,152]. Survival strategies of *P. gingivalis* result in remodeled microbiota which leads to a dysbiotic state that causes inflammatory disease such as periodontitis [16,145]

## 4. Clinical Implication of Periodontal Infections Caused by *P. gingivalis* on the Development of Coronary Artery Diseases

Numerous studies have established that people suffering from cardiovascular diseases have statistically significantly worse oral health than the general population. Accordingly, based on published data, it is evident that chronic periodontal disease increases the risk of coronary artery disease by 25% compared to healthy people [153]. The primary factor involved in the pathogenesis of atherosclerosis is inflammation. Therefore, chronic periodontal inflammatory disease is considered to be a risk factor for the progression of atherosclerosis. Namely, chronic periodontitis, which also has periods of an acute phase, causes a small but long-lasting systemic inflammatory reaction which contributes to the development of atherosclerosis [153]. Many periodontal pathogens have been found in human atherosclerotic plaques [154,155]. However, the most dominant bacteria in dentistry is *P. gingivalis*, which is the most frequently detected bacterium in patients’ atherosclerotic plaques [156].

**Figure 3:**
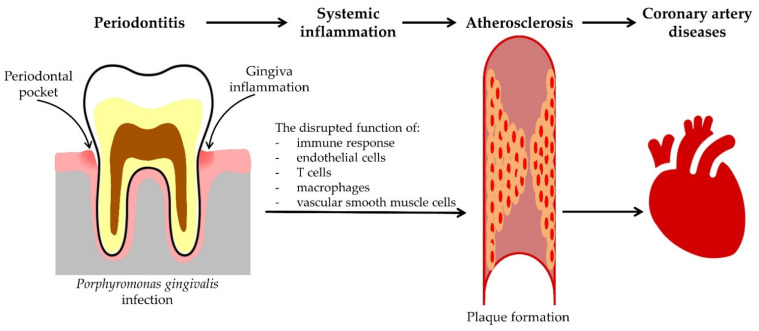
Association between periodontitis caused by *Porphyromonas gingivalis* infection, atherosclerosis, and coronary artery disease development.

Dental plaque microorganisms and their products, especially in advanced forms of the disease, can reach the blood during various therapeutic procedures (scaling, surgical procedures) and can reach distant organs through the blood. This primarily refers to the influence of periodontal pathogens on the development of subacute endocarditis, the development of coronary heart disease, atherosclerosis, and ischemic infarction [7,157] (Figure 3). Bacteremia of oral origin as a source of microorganisms can damage the heart valves causing bacterial endocarditis [158]. Among others, *P. gingivalis* is a risk factor for prothrombotic status that might worsen in patients with atrial fibrillation [159].

Furthermore, studies in mice have shown that *P. gingivalis* infection adversely affects the myocardium and promotes cardiomyocyte apoptosis [160,161]. Moreover, in vitro studies have shown that *P. gingivalis* LPS stimulates vascular calcification by affecting the expression of the osteogenic gene [162]. In another in vitro study, it was suggested that infecting primary human aortic endothelial cells with *P.gingivalis* can cause significant changes in the endothelial glycogen synthase kinase-3 beta, endothelial nitric oxide synthase, tetrahydrobiopterin, and nuclear factor erythroid 2–related factor pathways, which may lead to impaired vascular relaxation [163].

Based on knowledge about the connection between periodontitis and atherosclerosis, where ‘periodontal syndrome’ is considered an indicator of general health risk that should be taken care of, with a particular emphasis on developing the awareness of dentists and patients about this problem as a preventive measure.

### Atherosclerosis

Chronic periodontitis, which has periods of the acute phase, is thought to cause a small but long-lasting systemic inflammatory response, which contributes to the development of atherosclerosis and an increased risk of cardiovascular disease [24].

In endothelial cells, *P. gingivalis* induces dysfunction, destroys the integrity of the endothelium, and potentiates atherosclerotic plaque [164]. Endothelial dysfunction includes leakage of vascular endothelium that may be exacerbated by hypertension, dyslipidemia, or proinflammatory mediators, resulting in endothelial occlusion followed by more circulating low-density lipoprotein (LDL) [165].

*P. gingivalis* causes endothelial oxidative stress, and it is known that oxidative stress is a fundamental factor in the development of atherosclerosis [156]. *P. gingivalis*–gingipains specific for lysine (Kgp) and arginine (Rgp), which act as proteolytic enzymes and are responsible for antioxidant consumption and lipid peroxidation, contribute to the increase in oxidative stress. Antioxidant consumption occurs when *P. gingivalis* cleaves antioxidants in whole blood, causing a reduction in antioxidant levels. For example, cleavage of apoB100 by Rgp gingipain leads to aggregation of LDL and VLDL particles, a crucial process in forming an atherosclerotic plaque [166]. It destroys endothelial function by significantly increasing the production of superoxide free radicals and total reactive oxygen species (ROS). The signal axis to initiate this process is TLRs–NF–kB [156]. Subsequent oxidative stress is triggered by TLR participating in recognizing *P. gingivalis* LPS, followed by activation of the downstream signaling pathway NF–κB and its active subunit [167]. The resulting epithelial cell infection caused by *P. gingivalis* activates signaling cascades that control the transcription of target genes encoding the immune response and inflammatory responses such as interleukin (IL)-1β, IL-6, IL-8, and TNF-α in epithelial cells, monocytes, and interferon regulatory factor [167,168,169,170]. Additionally, *P. gingivalis* has the ability to enhance the regulation of CD36 belonging to the group of low-density lipoprotein (LDL) scavengers and oxidize them (oxLDL), resulting in the accumulation of oxLDL in macrophages. Thus, *P. gingivalis* LPS has a potential role in inducing macrophages to modify native LDL [171]. Likewise, *P. gingivalis* can initiate the oxidation of high-density lipoprotein (HDL), reverse the cholesterol transport pathway, remove cholesterol from macrophages, and thus contribute to atherosclerosis [172,173]. In addition, it can lead to an imbalance of proatherogenic and atheroprotective cytokines by disturbing the balance of Th17 and Treg [174]. Along with several other pathogens, *P. gingivalis* can convert macrophages into foam cells [175]. They earned their name because of their characteristic foamy appearance due to a load of cells with lipids [176]. It is precisely this ability to transform macrophages into foam cells that is associated with the accelerated development of atherosclerosis. All of this, as stated above, can induce proinflammatory changes in blood vessels [156,177]. In vivo studies on atherosclerosis-prone animals demonstrated that bacterial infection with *P. gingivalis* and other red complex bacteria results in accelerated coronary or aortic atherosclerosis and lipid deposition. Stronger expression of adhesion molecules (intercellular adhesion molecule-1 and vascular cell adhesion protein-1), toll-like receptor-4, and the lectin-like oxidized low-density lipoprotein receptor-1 was documented after venous injection of recombinant *P. gingivalis* heat-shock protein GroEL into B57BL/6 in mice [178]. In vitro studies on primary mouse brain endothelial cells showed that *P. gingivalis* upregulates IL-1β and TNF-α protein expression, which causes cell death of brain endothelial cells through the ROS/NF-κB pathway [179].

## 5. Conclusions

Coronary artery disease remains the leading cause of death in the general population, and a significant factor in the pathogenesis of coronary artery disease is inflammation caused by atherosclerotic plaques. An important factor in developing atherosclerotic plaque, among other periodontal pathogens, is *P. gingivalis*, which can significantly increase the risk and progression of cardiovascular disease. In addition, the interactions between oral and systemic diseases have been proven based on numerous studies. Therefore, to improve health care, it is necessary to continue further research.

## Figures and Tables

**Table 1:** *Porphyromonas gingivalis* virulence factors and its function.

Virulence Factors	Function	Ref
Fimbriae	Fimbriae promote biofilm formation, bacterial motility, adhesion, and invasion of host cells	[2,39,40]
Hemolysin	Serves to supply heme	[48]
Hemagglutinin	Serves to supply heme	[48]
Capsule	Encapsulation is associated with increased resistance to phagocytosis	[2,21]
Outer membrane vesicles (OMVs)	They are involved in the adaptation of bacteria to stress, nutrient metabolism, and communication with other periodontogens, and also host cells	[49]
Lipopolysaccharides (LPS)	Have the ability to cause an inflammatory reaction	[2]
Gingipains	Make up 85% of total proteolytic activity	[2,29,50]

## Data Availability

Not applicable.
